# Dietary patterns and associated factors of children under two years of age born prematurely

**DOI:** 10.1590/1984-0462/2022/40/2021177IN

**Published:** 2022-05-20

**Authors:** Ana Paula Kulig Godinho, Amanda de Oliveira da Conceição, Elisa Leite Rodrigues, Ilanna Mirela Becker Jorge Siqueira, Cesar Augusto Taconeli, Sandra Patrícia Crispim, Marcia Regina Messaggi Gomes Dias, Claudia Choma Bettega Almeida

**Affiliations:** a Universidade Federal do Paraná, Curitiba, PR, Brazil.

**Keywords:** Infant, premature, Complementary feeding, Food consumption, Dietary pattern, Feeding behavior, Recém-nascido prematuro, Alimentação complementar, Consumo alimentar, Padrões alimentares

## Abstract

**Objective:**

To identify the dietary patterns and associated factors of children aged between 6 and 23 months, born prematurely and assisted at a University Hospital in Curitiba, state of Paraná, Brazil.

**Methods:**

The parents or guardians of the 135 children were asked about their children’s eating habits and the family’s socioeconomic and demographic conditions. Information regarding birth and health history were obtained from medical records. Data on food consumption were subjected to exploratory factor analysis and the principal component analysis method was used to estimate the factor loads. Multiple linear regression was performed to verify possible associations.

**Results:**

Two dietary patterns were observed: “unhealthy” and “healthy.” The “unhealthy” pattern was significantly associated with maternal age, the child’s corrected age, and gestational age at birth. The “healthy pattern” was associated with the child’s corrected age. Maternal age and child’s corrected age remained significant after multiple regression analyses. For the “unhealthy” pattern, a positive effect was observed, suggesting that the consumption of this pattern is higher as the child’s age increases and less intense for children with mothers aged 30 years or older. For the “healthy” dietary pattern, the same two variables showed statistical significance. The authors observed a direct proportion between the age and consumption of food groups in both patterns.

**Conclusions:**

These results indicate the importance of nutritional education for younger mothers regarding their children’s eating practices, especially as the child grows.

## RESUMO

**Objetivo:**

Identificar os padrões alimentares e fatores associados de crianças entre seis e 23 meses de idade, nascidas prematuramente e assistidas em um Hospital Universitário de Curitiba, Paraná.

**Métodos:**

Os pais ou responsáveis pelas 135 crianças foram questionados quanto aos hábitos alimentares dos filhos e as condições socioeconômicas e demográficas da família. Informações relativas ao nascimento e ao histórico de saúde foram obtidas dos prontuários. Os dados de consumo alimentar foram submetidos a análise fatorial exploratória e o método dos componentes principais foi utilizado para estimar as cargas fatoriais. Regressão linear múltipla foi realizada para verificar possíveis associações.

**Resultados:**

Foram observados dois padrões alimentares distintos. Aquele denominado de “não saudável” apresentou associação significativa com a idade materna, a idade corrigida da criança e a idade gestacional ao nascer. Por sua vez, o padrão denominado de “saudável” esteve associado à idade corrigida da criança. As variáveis idade materna e idade corrigida da criança permaneceram significativas após as análises de regressão múltipla. Para o padrão “não saudável”, observou-se um efeito positivo, o que sugere que seu consumo é maior conforme a idade da criança aumenta e menos intenso para crianças com mães de 30 anos de idade ou mais. Para o padrão “saudável”, as mesmas duas variáveis mostraram significância estatística. Observamos uma proporção direta entre idade e consumo dos grupos alimentares em ambos os padrões.

**Conclusões:**

Esses resultados apontam para a importância da educação nutricional de mães mais jovens quanto às práticas alimentares de seus filhos, especialmente à medida que a criança cresce.

## INTRODUCTION

The number of preterm births, characterized by birth before the 37^th^ gestational week, has significantly increased in recent decades. Global estimates indicate approximately 15 million per year and one million infant deaths due to prematurity.^1^ Considered the main cause of death for children under five years of age, preterm birth is related to social inequalities and the absence of public policies that ensure basic care for the newborn such as support for breastfeeding.^1,2^

Due to the physiological risks and the deficit in caloric-protein storage resulting from prematurity, preterm babies demand greater nutritional support, which varies according to age. Thus, nutrition represents one of the fundamental pillars for the child’s survival.^2,3^ For preterm newborns, breastfeeding provides additional benefits. In addition to having immunological properties, breast milk helps to reduce the incidence and severity of necrotizing enterocolitis, sepsis and retinopathy of prematurity; it also assists in neuropsychomotor performance, in the lowest number of hospitalizations, and in the shortest hospital stay.^4,5^

For the onset of complementary feeding, the conditions of physiological and neuromuscular maturity, normally observed at six months of age in full-term babies, may not be present in premature babies.^6^ The act of expelling food with the tongue, tongue protrusion, gastrointestinal maturation, and the support capacity of the neck and chest are factors that allow the safe swallowing of food and they must be observed. Another important factor is to consider the child’s corrected age (CA).^2,5^ Also known as postconceptional age, CA consists in the adjustment of chronological age in relation to the degree of prematurity, when considering the ideal gestational age (GA) of 40 weeks.^7^

Due to the fact that chronological age does not determine the onset of the offer of new foods, the challenges of complementary feeding for children born prematurely are numerous. Thus, identifying the children’s dietary patterns can contribute to the creation of nutritional strategies. A dietary pattern represents a set of foods frequently consumed by a population, enabling the diet to be assessed from a global perspective.^8^ This approach considers cultural, demographic, and socioeconomic aspects that can interfere with food choice.^9,10^

Taking this into consideration and the lack of research with a similar approach on this population, the present study aimed to identify the dietary pattern of children born prematurely and the associated factors.

## METHOD

This is a cross-sectional study that evaluated the complementary feeding practices of children born prematurely and assisted at the outpatient clinic for monitoring newborns at risk of a University Hospital in the city of Curitiba, state of Paraná, Brazil. About 100 children are attended per week, including medical, nursing, and social assistance appointments. The outpatient clinic does not offer consultations with a professional nutritionist.

Data collection was carried out from May 2018 to April 2019, after approval by the Ethics Committee of Complexo Hospital de Clínicas, Universidade Federal do Paraná (CHC-UFPR), No. 2.568.960, and the signing of the informed consent form by the parents or guardians of the participating children. The sample was defined by convenience and consisted of children aged between six and 23 months, born prematurely (<37 gestational weeks). Those who had severe congenital malformation or neurological disease, those who were using tubes or ostomy for feeding, those whose parents or guardians were absent the day before the interview, and those who lived in shelters were excluded.

The instrument used for data collection was elaborated in two parts and developed based on the document called “Postnatal Infant Follow-up Study,” from the University of Oxford.^11^ In the first part, parents or guardians were asked about the children’s eating habits and the socioeconomic and demographic conditions of the family. Data on the birth and health history of the children were obtained from medical records.

Based on the description of food consumption on the day before the assessment, food and beverages were recorded in quantities, preparation and presentation, according to the consumed meals. For the application of the 24-hour recall (R24h), the Multiple Pass Method (MPM) was considered, consisting of five steps: (1) quick list of foods; (2) list of commonly forgotten foods; (3) definition of time and occasion; (4) detail cycle; and (5) final probe, seeking to assist in the interviewee’s detailed memory process.^12^

**Chart 1:**
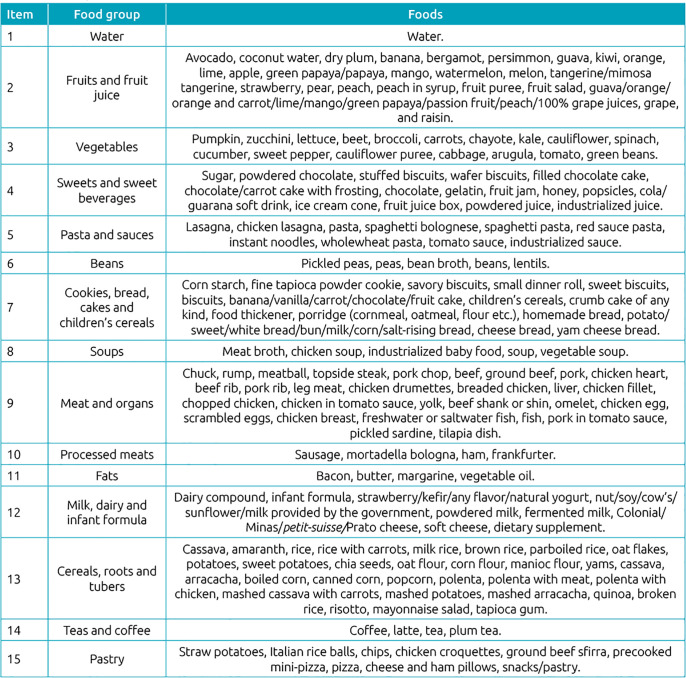
Food groups formed with foods listed in the 24-hour recalls and their respective items.

Food consumption data were entered into the REC24H-ERICA software. The described portions were initially standardized and converted into grams or milliliters. The conversion of household measures was carried out based on the Table of Reference Measures for Food Consumed in Brazil from the Brazilian Household Budget Survey (*Pesquisa de Orçamentos Familiares* – POF) 2008/2009^13^ and on the information present on labels of the products. The mentioned foods were grouped according to their nutritional similarity and frequency of consumption, totaling 15 groups named according to composition. The formed groups and their respective foods are shown in Chart 1.

Descriptive statistical analysis was performed for categorical variables by calculating absolute (n) and relative (%) frequencies. In the case of the continuous variable (maternal age), the median was calculated with their respective quartiles. For the assessment of the dietary pattern, the *a posteriori* approach was used. This exploratory method employs multivariate analysis techniques to derive dietary patterns based on the observed consumption. This type of analysis allows aggregating the foods consumed by the individual and reducing them to smaller data sets that represent exposure to the diet.^9,10,14,15^

Data were subjected to exploratory factor analysis and the Principal Component Analysis method was used to estimate factor loads and obtain commonalities and specificities. The number of factors (patterns) retained in the analysis was determined by simulation using parallel analysis, in which the eigenvalues of the sample correlation matrix were compared with the corresponding eigenvalues produced by completely-randomly generated data. A cutoff point of 0.25 was defined to assist in the interpretation of factors, but all values were considered for the calculation of factor scores. The Kaiser-Meyer-Olkin (KMO) index and the ratio of cases per variable were applied to assess the adequacy of the sample to factor analysis.^16,17^

A varimax-type rotation was applied to produce a better definition of dietary patterns. Factor scores were calculated for each individual in the sample, based on the factor loads corresponding to each pattern and the respective responses. Based on the factor scores obtained, a multiple linear regression model was adjusted to identify possible associations with characteristics of the family, mother, or the very child. All analyses were performed using the R statistical software, version 3.6.3, and the psych library was used in all stages of the exploratory factor analysis.

## RESULTS

A total of 135 children participated in the study, with a predominance of girls (54.8%; n=74). The corrected age median was ten months (25^th^ percentile [p25]=7; 75^th^ percentile [p75]=14), with the highest frequency in the age group between six and 11 months (53.3%; n=72). Most were born moderately preterm (66.7%; n=90) and with adequate weight for gestational age (75.5%; n=102). A total of 87% (n=117) of the children had some neonatal complication, and gastrointestinal (77.8%; n=91) and respiratory (62.2%; n=84) complications were the most common. The median length of hospital stay was 20 days (p25=8; p75=50). The complete characterization of children is presented in Table 1.

**Table 1.** Demographic and health characteristics of children under 24 months of corrected age, born prematurely (n=135).

Characteristics	%	n
Sex		
Male	45.18	61
Female	54.82	74
Corrected age (months)		
<6	8.15	11
6<12	53.33	72
≥12<18	22.97	31
≥18	15.55	21
Gestational age at birth (weeks)		
<28	9.63	13
28<32	23.70	32
≥32	66.67	90
Weight for gestational age*		
Small	17.78	24
Adequate	75.55	102
Large	6.67	9
Neonatal complications		
Yes	86.67	117
No	13.33	18
Type of neonatal complication**		
Respiratory	62.22	84
Hepatic/gastrointestinal	67.4	91
Cerebral/neuromuscular	14.07	19
Cardiac/circulatory	9.62	13
Renal	5.18	7
Sepsis	20.0	27
Others (ophthalmologic, metabolic)	36.29	49
Length of hospital stay (days)		
≤20	51.11	69
>20	48.89	66

*According to the growth curves of the World Health Organization (2006); **the sum of individuals exceeds the total number of the sample, as some children had more than one type of neonatal complication.

Regarding mothers, the median age was 31.5 years (p25=23; p75=36), and 41.5% (n=46) of them had an income between 0.5 and 1.0 minimum wage per capita. Most mothers self-reported to be white (65.7%; n=88), having more than eight years of formal education (90.3%; n=94), living with their spouse (85.2%; n=115), having more than one child (53.3%; n=72), and not working outside the home (67.4%; n=91). As for the children’s diet, 24.4% (n=33) continued to receive breast milk in addition to complementary feeding. The median duration of breastfeeding was 135 days (p25=2; p75=240). The mothers’ characteristics are shown in Table 2.

**Table 2.** Mothers’ sociodemographic and health characteristics (n=35).

Characteristics	%	n
Age* (years)		
<20	8.33	11
20–35	60.61	80
>35	31.06	41
Race/skin color**		
White	65.67	88
Black	7.46	10
Mixed-race	21.64	29
Other (Asian, Indigenous)	5.23	7
Level of education (years)		
<4	1.48	2
4–8	8.15	11
>8<12	69.63	94
≥12	20.74	28
Work		
Yes	32.59	44
No	67.41	91
Income per capita (minimum wage)†		
<0.25	7.41	10
0.25<0.50	28.89	39
≥0.50<1.00	41.48	56
≥1.00	22.22	30
Lives with the spouse		
Yes	85.19	115
No	14.81	20
Previous pregnancies		
Primiparous	46.67	63
Multiparous	53.33	72

*Not informed=3 (2.2%); **Not informed=1 (0.7%); †minimum wage value at the time of data collection: BRL 954.00/USD 242.13.

More than half of the children (53.3%; n=72) were already consuming another type of milk (cow’s or soy-based milk) other than breast milk, being nine months old (p25=7; p=75=11.7) the median age at the onset of this practice. The introduction of food in 66.6% (n=90) of the children occurred before six months of corrected age, with five months (p25=4; p75=6) being the median age. As for liquids, 80.4% (n=108) had already consumed some type of beverage before six months of age, with the median age of onset being four months (p25=3; p75=5).

According to exploratory factor analysis, only two dietary patterns were verified. Three food groups (“pastry,” “soups,” and “processed meats”) showed commonality less than 0.2, indicating that less than 20% of the original variation of these groups was explained by the two dietary patterns obtained. The authors decided to remove them and repeat the analysis considering the others. The estimated KMO index was equal to 0.74, indicating the factor analysis as appropriate for the present study. Table 3 presents the summary of the resulting exploratory factor analysis.

**Table 3.** Summary of the exploratory factor analysis.

Group	Unhealthy	Healthy	Commonality	Specificity
Sweets and sweet beverages	0.29	0.01	0.48	0.52
Water	-0.13	-0.21	0.30	0.70
Cookies, bread, cakes and children’s cereals	0.35	0.04	0.51	0.49
Teas and coffee	0.21	0.03	0.33	0.67
Meat and giblets	0.11	0.31	0.43	0.57
Milk, dairy and infant formula	0.33	0.07	0.46	0.54
Beans	0.05	0.31	0.40	0.60
Fruits and fruit juice	-0.15	0.26	0.39	0.61
Fats	0.19	0.02	0.34	0.66
Vegetables	0.07	0.31	0.40	0.60
Pasta and sauces	0.24	0.06	0.31	0.69
Cereals, roots and tubers	0.01	0.29	0.45	0.55
% variance	21	20		
% accumulated variance	21	41		

Table 4 presents the bivariate descriptive analyses for the “unhealthy” and “healthy” patterns. The consumption of the “unhealthy” pattern was significantly associated with maternal age, child’s CA, GA at birth, and with the fact that the mother lives with her spouse. As for the “healthy pattern,” only the child’s CA produced a significant difference. Variables that produced p<0.20 in the bivariate analysis were later considered in the multiple regression analysis.

**Table 4.** Distribution of dietary pattern scores according to socioeconomic and demographic variables.

	n	Unhealthy		Healthy	
		Mean (SD)	p-value	Mean (SD)	p-value
Maternal age (years)					
<20	11	1.25 (0.98)	<0.001	-0.60 (0.98)	0.102
20–35	83	-0.11 (0.91)		0.03 (1.02)	
>35	41	-0.12 (0.97)		0,11 (0.94)	
Mother’s level of education (years)					
≤8	13	0.35 (1.31)	0.188	0.12 (1.15)	0.648
≥8	122	-0.04 (0.96)		-0.01 (0.99)	
Lives with the spouse					
Yes	115	-0.12 (0.94)	0.001	0.05 (0.96)	0.176
No	20	0.70 (1.06)		-0.28 (1.17)	
First child					
Yes	63	0.02 (1.14)	0.796	-0.14 (1.08)	0.139
No	72	-0.02 (0.87)		0.12 (0.92)	
Work					
Yes	44	-0.11 (0.94)	0.364	0,19 (0.94)	0.127
No	91	0.05 (1.03)		-0.09 (1.02)	
Gestational age at birth					
<28 weeks	13	0.75 (1.18)	0.016	0.35 (0.91)	0.216
28–31 weeks	32	-0.08 (0.94)		0.14 (0.97)	
≥32 weeks	90	-0.08 (0.96)		-0.10 (1.02)	
Child’s corrected age					
<6 months	11	-1.08 (0.46)	<0.001	-0.21 (0.48)	0.001
6–11 months	72	-0.28 (0.80)		-0.28 (1.01)	
12–17 months	31	0.56 (0.91)		0.47 (0.86)	
≥18 months	21	0.68 (1.10)		0.36 (1.04)	
Breastfeeding					
Yes	32	-0.28 (0.88)	0.067	0.17 (0.85)	0.249
No	102	0.09 (1.02)		-0.06 (1.04)	
Breastfeeding duration					
<135 days	69	0.01 (1.01)	0.867	-0.08 (0.97)	0.325
≥135 days	66	-0.01 (1.00)		0.09 (1.03)	
Onset of complementary feeding					
<6 months	90	-0.03 (0.99)	0.612	-0.06 (0.95)	0.469
6 months	31	-0.02 (1.12)		0.03 (1.11)	
>6 months	14	0.25 (0.79)		0.30 (1.07)	

SD: standard deviation

Table 5 presents the summary of the resulting model for the “unhealthy” and “healthy” dietary patterns. For the “unhealthy” pattern, there was a positive effect associated with the child’s CA. This suggests that consumption of the “unhealthy” pattern is higher as the child’s age increases. Regarding maternal age, the consumption of the “unhealthy” pattern was less intense for children whose mothers were 30 years old or more compared with mothers under 20 years old. For the “healthy” dietary pattern, the same two variables showed statistical significance.

**Table 5.** Multiple linear regression model adjusted to dietary pattern scores.

Unhealthy				
Parameter	Estimation	Standard error	Student’s t-test	p-value
Intercept	0.03	0.35	0.09	0.924
Maternal age				
20–34 years	1.10	0.26	-4.22	<0.001
≥35 years	1.22	0.27	-4.47	<0.001
Child’s corrected age				
6–11 months	0.75	0.26	2.86	0.004
12–17 months	1.54	0.28	5.46	<0.001
≥18 months	1.64	0.30	5.36	<0.001

Healthy				
Parameter	Estimation	Standard error	Student’s t-test	p-value
Intercept	-0.97	0.41	-2.36	0.019
Maternal age				
20–34 years	0.75	0.30	2.49	0.013
≥35 years	0.82	0.31	2.60	0.010
Child’s corrected age				
6–11 months	0.03	0.30	-0.11	0.912
12–17 months	0.74	0.32	2.25	0.025
≥18 months	0.65	0.35	1.83	0.068

## DISCUSSION

The present study identified two opposing dietary patterns. The first, named “unhealthy,” was characterized by the presence of foods with high energy density and low nutritional quality, and the second, named “healthy,” was composed of food groups recommended for an adequate complementary feeding. It was observed that older mothers tend to offer healthier foods to their children and that, as the child grows, they start consuming more foods deemed unhealthy.

The onset of complementary feeding in more than half of the children occurred before six months of CA. Similar results were reported in an Italian cohort, in which the mean CA at the time of introduction of complementary foods was 4.6 months among 146 babies born prematurely in a hospital in Milan.^18^ Also in Italy, another cohort study had already identified the early introduction of complementary foods around the 15^th^ week of corrected age. Among the associated factors, younger maternal age significantly influenced early food introduction.^19^

Although the main health agencies recommend exclusive breastfeeding (EBF) until the sixth month of life and the introduction of new foods from that age onwards, many pediatricians recommend the offer of food, especially fruits, before the age of six months, particularly in cases in which the mother works outside the home. This would consist in a way to make the transition between EBF and complementary feeding while still on maternity leave.

In the “unhealthy” pattern, it was possible to identify foods, such as cookies and sweets, characterized by high energy density and low nutritional quality, not recommended for children under two years of age.^6,20^ In a study carried out in a maternity hospital in the state of Rio de Janeiro, Brazil, with 108 preterm babies, wheat-based foods, cow’s milk, and ultra-processed foods were highlighted as the main factors interfering in the adequacy of the babies’ diet.^21^

In the present study, the “healthy” dietary pattern included fresh and minimally processed foods, following the recommendations of national and international health agencies regarding healthy eating practices.^6,20,22^ The first two years of a child’s life are essential to determine their growth and development.^23^ Thus, a balanced diet is capable of optimizing growth, body composition, neurological development, and even the infant gut microbiota, also in regard to premature children.^23,24,25,26^

Similar results were obtained by a study conducted by Salles-Costa et al.^27^ The researchers identified three dietary patterns in a sample of 366 children under 30 months of age and residents of the city of Duque de Caxias, state of Rio de Janeiro, Brazil. Among the associated factors, lower maternal age represented the greatest chances of the child belonging to the “unhealthy” pattern. Internationally, a study with 486 Asian children that detected four dietary patterns in the first year of life associated younger maternal age with the consumption of the “easy-to-prepare foods” pattern, consisting of products deemed unhealthy.^28^

The authors of the present study observed a direct proportion between the age and consumption of food groups in both patterns. This result was already expected, as it is natural for older children to be more exposed to different types of food due to their greater physiological aptitude and greater security on the part of parents or guardians.^6,20^

Although no significant association between maternal education and dietary patterns was found, a joint report released by the Pan American Health Organization and World Health Organization (PAHO/WHO), the United Nations Children’s Fund (UNICEF), and the United Nations Population Fund (UNFPA)^29^ reported that 22.4% of preterm births occurred in women under 20 years of age. As a chain reaction, many girls who become pregnant at a young age drop out of school. The negative consequences from an educational, professional, financial, political, and social point of view configure a vicious cycle, in which the lack of education can justify the reproduction of unhealthy eating practices in children. In this sense, the study by Souza et al.^30^ identified greater adherence to the “vegetables and fruits” pattern in children under five years of age, whose mothers had higher levels of education.

Nonprobability sampling and the instrument used to assess food consumption may characterize limitations of the present study. Although it is not possible to measure the accuracy of the results using statistical tools, in the universe of children assisted by the high-risk newborn outpatient clinic, only those born prematurely and who met the inclusion criteria of the study were selected, representing adequate sources of information for the proposed objectives. As for the instrument for assessing food consumption, obtaining verbal information depends on the respondent’s cooperation, communication skills, and memory, and may not reflect their eating routine. Nevertheless, children’s diet during the first two years of life is generally characterized by low diversity. Furthermore, the MPM was used seeking to assist in the detailed memory process of parents or guardians.

This study identified two dietary patterns with opposite nutritional characteristics. The first is characterized by the presence of foods with high energy density and low nutritional quality, and the second includes fresh and minimally processed foods, recommended for an adequate complementary feeding. Maternal age and child’s CA were associated with both verified patterns. It was observed that older mothers tend to offer healthier foods to their children. Conversely, as children grow, consumption of foods considered unhealthy increases.

In addition to dietary aspects, the results of this study point to the importance of nutritional education, especially during childhood and adolescence. Without actions that promote adequate and healthy eating, the reproduction of unhealthy eating practices, which pass on from generation to generation, is justified. With regard to premature children, inadequate eating practices represent even greater risks of malnutrition compared with full-term children.
